# Draft Genome Sequences of a *Bifidobacterium* Strain and a *Bacteroides* Strain Isolated from a Human Stool Sample

**DOI:** 10.1128/mra.00011-22

**Published:** 2022-03-23

**Authors:** Woojung Shin, Hyun Jung Kim

**Affiliations:** a Department of Biomedical Engineering, The University of Texas at Austin, Austin, Texas, USA; b Department of Oncology, Dell Medical School, The University of Texas at Austin, Austin, Texas, USA; c Wyss Institute for Biologically Inspired Engineering, Harvard University, Boston, Massachusetts, USA; University of Maryland School of Medicine

## Abstract

We present draft genome sequences of Bifidobacterium longum subsp. *longum* JCM 7050 and *Bacteroides* sp. strain 1_1_30 isolated from a healthy donor’s fecal sample obtained from a public stool bank, OpenBiome. Phylogenetic and functional analyses were performed to understand the physiological characteristics and functions of the strains in the human intestine.

## ANNOUNCEMENT

Here, we announce draft genome sequences of a Bifidobacterium longum strain and a *Bacteroides* strain isolated from a processed fecal sample obtained from a public stool bank, OpenBiome (1). The fecal sample, which qualified for fecal microbiota transplantation, was obtained from a donor who met the screening criteria without risk factors for infection or microbiome-associated health conditions. Detailed information on the criteria is available from the OpenBiome Quality and Safety Program (2).

The processed frozen fecal sample was used to isolate single microbial strains. After thawing a vial of frozen fecal slurry at room temperature for 10 min, we performed single-colony isolation by streaking the slurry on a De Man-Rogosa-Sharpe (MRS) agar plate containing mupirocin (50 mg/L) and l-cysteine (0.05% [wt/vol]). The agar plate was incubated for 24 h at 37°C in an anaerobic glove box (90% N_2_, 5% CO_2_, and 5% H_2_). A single microcolony was selected for subculture on a new agar plate and was cultured for 24 h at 37°C under the same anaerobic conditions. The microbial cells grown from the isolated colony were subcultured for 24 h at 37°C in a test tube with 3 mL of MRS broth containing mupirocin (50 mg/L) and l-cysteine (0.05% [wt/vol]) in the anaerobic chamber without shaking. We repeated this process to isolate independent colonies. After enrichment of individual isolates, the culture broth was mixed with sterilized glycerol (20% [vol/vol] final concentration) and stored at −80°C.

Bacterial genomic DNA isolated from a frozen stock was purified using a QIAamp PowerFecal DNA kit (Qiagen) following the manufacturer’s protocol, quantified using a NanoDrop One microvolume UV-visible spectrophotometer (Thermo Fisher Scientific), and then submitted for whole-genome sequencing (WGS) (CosmosID). The Ion Xpress Plus fragment library kit (Thermo Fisher Scientific) was used to produce libraries, which were subsequently sequenced using the Ion S5 next-generation sequencing system (Thermo Fisher Scientific) with a read length of 151 bp. Table 1 summarizes genome assembly statistics and strain information. BBDuk (BBMap v36.49) was used to trim and process the raw single-end reads, with a read quality trimming setting of 20 (3). The SPAdes assembler v3.9.0 was used to complete the genome assembly (4). The lineage_wf function in CheckM v1.0.13 was used to assess the completeness of the constructed isolates (5). The National Center for Biotechnology Information (NCBI) Prokaryotic Genome Annotation Pipeline (PGAP) was used to annotate the assembled isolate genomes with the best-placed reference protein set method (GeneMarkS-2+ v5.3) (6–8). Unless otherwise indicated, default parameters were utilized for all software.

**TABLE 1:** Genome assembly statistics and strain information

Strain	GenBank accession no.	SRA accession no.	Total no. of reads	Genome coverage (×)	Avg contig coverage (×)	No. of contigs	No. of genes	No. of coding sequences	Genome size (bp)	No. of tRNAs	GC content (%)	*N*_50_ (bp)
B. longum subsp. *longum* JCM 7050	JAJKHM000000000	SRX13589959	3,485,108	230.03	29,902.29	76	1,960	1,899	2,272,574	55	60.0	68,170
*Bacteroides* sp. strain 1_1_30	JAJKHN000000000	SRX13589958	5,814,308	133.85	24,773.51	263	5,319	5,255	6,515,432	59	44.7	62,558

The WGS of the isolates revealed that B. longum subsp. *longum* JCM 7050 and *Bacteroides* sp. strain 1_1_30 have genome sizes of 2,272,574 and 6,515,432 bp, respectively, with GC contents of 60.0% and 44.7%. Functional annotations of the assembled genome demonstrated 1,899 total coding sequences and 55 tRNA sequences for B. longum subsp. *longum* JCM 7050 and 5,255 total coding sequences and 59 tRNA sequences for *Bacteroides* sp. strain 1_1_30.

### Data availability.

The whole-genome shotgun sequences of B. longum subsp. *longum* JCM 7050 and *Bacteroides* sp. strain 1_1_30 were deposited in GenBank under accession numbers JAJKHM000000000 and JAJKHN000000000, respectively, and the raw reads can be found as Sequence Read Archive (SRA) data with accession numbers SRX13589959 and SRX13589958, respectively, under BioProject accession number PRJNA761794.
